# Author Correction: Heat transfer analysis of Cu and Al_2_O_3_ dispersed in ethylene glycol as a base fluid over a stretchable permeable sheet of MHD thin-film flow

**DOI:** 10.1038/s41598-022-16501-y

**Published:** 2022-07-26

**Authors:** Zeeshan, Ilyas Khan, Wajaree Weera, Abdullah Mohamed

**Affiliations:** 1 Department of Mathematics and Statistics, Bacha Khan University Charsadda, Charsadda, KP Pakistan; 2 Department of Mathematics, College of Science Al‑Zulfi, Majmaah University, Al‑Majmaah, 11952 Saudi Arabia; 3 Department of Mathematics, Faculty of Science, Khon Kaen University, Khon Kaen, 40002 Thailand; 4 Research Centre, Future University in Egypt, New Cairo, 11835 Egypt

Correction to: *Scientific Reports* https://doi.org/10.1038/s41598-022-12671-x, published online 25 May 2022

In the original version of this Article Abdullah Mohamed was incorrectly affiliated with ‘Department of Mathematics, College of Science and Humanities in Al‑Afaj, Prince Sattam bin Abdulaziz University, Al‑Afaj 11942, Saudi Arabia’ and ‘Administration Department, Administrative Science College, Thamar University, Thamar, Yemen’. The correct affiliation is listed below.

Research Centre, Future University in Egypt, New Cairo 11835, Egypt.

The original Article has been corrected.

